# Akt2 Regulates Metastatic Potential in Neuroblastoma

**DOI:** 10.1371/journal.pone.0056382

**Published:** 2013-02-26

**Authors:** Jingbo Qiao, Sora Lee, Pritha Paul, Lan Qiao, Chase J. Taylor, Cameron Schlegel, Nadja C. Colon, Dai H. Chung

**Affiliations:** 1 Department of Pediatric Surgery, Vanderbilt University Medical Center, Nashville, Tennessee, United States of America; 2 Department of Cancer Biology, Vanderbilt University Medical Center, Nashville, Tennessee, United States of America; Van Andel Institute, United States of American

## Abstract

Activation of PI3K/AKT pathway correlates with poor prognosis in patients with neuroblastoma. Our previous studies have demonstrated that PI3K/AKT signaling is critical for the oncogenic transformations induced by gastrin-releasing peptide (GRP) and its receptor, GRP-R, in neuroblastoma. Moreover, PI3K/AKT-dependent oncogenic transformations require N-myc, an extensively studied oncogene in neuroblastoma. Whether AKT directly regulates the expression of N-myc oncogene is yet to be determined. Here, we report a novel finding that of the three AKT isoforms, AKT2 specifically regulated N-myc expression in neuroblastoma cells. We also confirmed that GRP-R is upstream of AKT2 and in turn, regulated N-myc expression via AKT2 in neuroblastoma cells. Functional assays demonstrated that attenuation of AKT2 impaired cell proliferation and anchorage-independent cell growth, and decreased the secretion of angiogenic factor VEGF *in vitro*. Furthermore, silencing AKT2 inhibited migration and invasion of neuroblastoma cells *in vitro*. Xenografts established by injecting AKT2 silenced human neuroblastoma cells into murine spleen expressed decreased levels of AKT2 and resulted in fewer liver metastases compared to controls *in vivo*. Hence, our study highlights the potential molecular mechanism(s) mediating the oncogenic role of GRP/GRP-R and demonstrates a novel role for AKT2 in neuroblastoma tumorigenesis, indicating that targeting the GRP/GRP-R/AKT2 axis may be important for developing novel therapeutics in the treatment of clinically aggressive neuroblastoma.

## Introduction

Neuroblastoma is a pediatric neuroendocrine tumor that can produce and secrete a variety of neuropeptides, including gastrin-releasing peptide (GRP) [1]. GRP binds to G-protein coupled receptor, GRP-R, to stimulate growth of a number of normal and neoplastic tissues of the gastrointestinal tract, including neuroblastoma [2]. In spite of recent advances in understanding the role of GRP/GRP-R in tumor progression [3]–[5], signal transduction pathways regulated by GRP and its receptor are not completely understood. We have previously reported that the PI3K/AKT pathway, in part, mediates GRP-induced G1-S phase cell cycle progression and that bombesin, an amphibian equivalent of GRP, induces vascularization of neuroblastoma xenografts by upregulation of vascular endothelial growth factor (VEGF) [3], [4]. Correspondingly, we also reported that GRP-R overexpressing neuroblastoma cells induce AKT activation, and the ratio of AKT to PTEN, an endogenous negative regulator of PI3K, is increased in neuroblastoma patients [5].

AKT activation has been shown to be an indicator of malignancy [6] and chemoresistance [7] in neuroblastoma. AKT kinase family is composed of three isoforms with different cellular functions. AKT1 regulates cell survival, and AKT3 plays a critical role in brain development [8], whereas, AKT2 is more important in cancer development and progression [9]–[12]. Interestingly, silencing GRP-R predominantly reduces AKT2 expression without affecting the expression of AKT1 or AKT3 isoform [13], thus demonstrating a more critical role of AKT2 in GRP-R-mediated neuroblastoma tumorigenesis. However, the exact role of AKT2 in neuroblastoma remains unclear.

*MYCN*, a critical oncogene in neuroblastoma, is amplified in approximately 25% of the cases and its amplification strongly correlates with poor outcomes in neuroblastoma patients [14]–[16]. Activation of PI3K/AKT pathway has been reported to stabilize N-myc protein by dephosphorylation in neuroblastoma cells [14]. We have previously reported that N-myc acts as a critical downstream effector of PI3K/AKT in neuroblastoma tumor angiogenesis [17]. Whether AKT isoforms directly regulate the expression of N-myc in neuroblastoma is unknown. Moreover, a role for GRP/GRP-R/AKT axis in the regulation of the *MYCN* oncogene in neuroblastoma is yet to be studied.

In this study, we identified a novel regulation of N-myc expression by the AKT2 isoform in neuroblastoma cells. We also demonstrate that GRP-R regulates AKT2-mediatd N-myc expression. Interestingly, silencing AKT2 decreases neuroblastoma cell proliferation, anchorage-independent growth, migration and invasion, and VEGF secretion *in vitro*. Moreover, intrasplenic injection of AKT2 silenced neuroblastoma cells decreased the formation of liver metastases *in vivo*. Hence, we demonstrate that studying the GRP-R/AKT2/N-myc signaling axis may provide novel insights into the pathobiology of neuroblastoma tumorigenesis.

## Materials and Methods

### Cells and cell culture

Human neuroblastoma BE(2)-C, BE(2)-M17, and SK-N-BE(2) cell lines were purchased from American Type Culture Collection (Manassas, VA). Cells were cultured in RPMI 1640 medium with L-glutamine (Cellgro Mediatech, Inc. Herndon, VA) supplemented with 10% fetal bovine serum (FBS, Sigma, St. Louis, MO). The cells were maintained at 37°C in a humidified atmosphere of 95% air and 5% CO_2_.

### Plasmids, siRNAs and transfections

Plasmid pCDNA3.1 was obtained from Invitrogen (Carlsbad, CA), and pcDNA3-Myr HA-AKT2 (Addgene plasmid 9016) was purchased from Addgene (Cambridge, MA). Plasmid pLKO.1-shAKT2 (human NM_001626) and its control vector SHC002 (shCON) were purchased from Sigma. siRNA pools targeting AKT1 (siAKT1), AKT2 (siAKT2), AKT3 (siAKT3), and non-targeting control siRNA (siNTC) were purchased from Dharmacon, Inc (Lafayette, CO). Human neuroblastoma cells were transfected with plasmids or siRNA using Lipofectamine 2000 (Invitrogen) as previously described [3], [5]. BE(2)-C/shCON and BE(2)-C/shGRP-R cells were stably transfected with shRNA as described [13]. Stably-transfected BE(2)-C/shAKT2 cells were selected with puromycin (Sigma) at 2.5 µg/ml for one week. GRP and IGF-1 were purchased from Bachem (Torrance, CA). GRP (100 nM) and IGF-1 (100 nM) stimulations were performed following overnight serum starvation, and samples were collected at the indicated time points.

### Inducible knockdown system

For the knockdown of our target genes, human GRP and GRP-R, we used BLOCK-iT Inducible H1 Lentiviral RNAi System (Life Technologies, Invitrogen. Grand Island, NY). The sequence targeting GRP-R (NM_005314) is underlined in the following shRNA (shGRP-R) sequence: 5′-CACCGTAACGTGTGCTCCAGTGGACGAATCCACTGG AGCACACGTTA-3′; the sequence targeting GRP (NM_002091) is underlined in the shRNA (shGRP) sequence: 5′-CACCAGCAATCAGCAGCCTTCGTGGGACGAATCCCACGAAGG CTGCTGATTGC-3′; the nonspecific control shRNA (shCON) is: 5′-CACCGGGCGCGCTTTGTAGGATTCGCC GAAGCGAATCCTACAAAGCGCGCC-3′. shRNA sequences were cloned into the BLOCK-iT Inducible H1 RNAi Entry Vector (pENTRTM/H1/TO). Then, shRNA was inserted into Lentiviral vector pLenti4/BLOCK-iT-DEST by LR recombination between pENTRTM/H1/TO entry and pLenti4/BLOCK-iT expression constructs. Inducible shRNA expression cells were established by transfecting BE(2)-C cells with both pLenti6/TR and pLenti4/BLOCK-iT-DEST, or by introducing the vectors with the lentiviral-mediated delivery system. Production of lentivirus was performed in 293FT cells. Stable cell lines BE(2)-C/Tet/shCON, BE(2)-C/Tet/shGRP, and BE(2)-C/Tet/shGRP-R were established by selecting with Blasticidin at 8 µg/ml and Zeocin at 50 µg/ml post lentiviral transductions.

### Cell proliferation and Soft agar colony formation assay

Cells were seeded onto 96-well plates at a density of 1×10^4^ cells per well in RPMI culture media with 10% FBS, and cell proliferation was assessed using Cell Counting Kit-8 (Dojindo Molecular Technologies, Rockville, MD). Cells were trypsinized and resuspended in RPMI 1640 containing 0.4% agarose (Cambrex Bio Science, Rockland, ME) and 5% FBS. Using a 12-well plate, cells were overlaid onto a bottom layer of solidified 0.8% agarose in RPMI 1640 containing 10% FBS at a concentration of 2×10^3^ cells/well and incubated for 3 weeks. Colonies were stained with 0.005% crystal violet dissolved in 70% methanol before being photographed and quantified.

### VEGF ELISA

The supernatant from cultured cells was collected at the indicated time points and VEGF levels were measured using human VEGF ELISA kit (R&D systems Inc. Minneapolis, MN) according to the manufacturer's instructions. All experiments were performed on at least two separate occasions.

### Migration and invasion assays

For transwell migration, transwell filters (8 µm; Corning, Lowell, MA) were coated on the lower chamber with 5 µg/ml collagen type I (BD Biosciences) overnight and then blocked with 2.5% BSA/PBS for 1 h. 1×10^5^ cells in serum-free media were added to the upper chamber and 10% FBS containing RPMI media into bottom well, and incubated for 6 h. Cells were fixed with 4% paraformaldehyde, stained with DAPI, and counted. For invasion assay, the transwell filters were coated with 1/30 diluted Matrigel (BD Biosciences). Cells (1.5×10^5^) in serum-free media were added to the upper well and 10% FBS containing RPMI media was added to the bottom well. After 48 h incubation, cells were fixed with 4% paraformaldehyde, stained with DAPI, and counted. Assay was performed in duplicate, and cells were counted from five randomly selected microscopic fields.

### Western blot and RT-PCR analyses

Primary antibodies against GRP-R (Abcam, Cambridge, MA), AKT1, AKT2, AKT3 and N-myc (Cell Signaling, Danvers, MA) were used. Horseradish peroxidase-conjugated secondary antibodies against mouse and rabbit IgG were obtained from Santa Cruz Biotechnology, Inc. (Santa Cruz, CA). β-actin antibody was from Sigma-Aldrich. Western blot analysis was performed to evaluate the efficacy of transfection with plasmid, shRNA, and siRNA in cells, while using β-actin as a loading control. Total RNA was isolated using the RNAqueous™ kit (Ambion, Austin, Texas) according to the manufacturer's instructions. Isolated RNA was used to synthesize cDNA using the High-Capacity cDNA Reverse Transcription Kit (Applied Biosystems, Austin, TX). The level of mRNA was measure by quantitative real-time PCR using SsoFast™ EvaGreen Supermix with CFX96 Real-Time PCR System (Bio-Rad, Hercules, CA). Primers designed to amplify a 192-bp MYCN fragment (NM_005378): forward primer 5′- GCTTCTACCCGGACGAAGATG-3′; reverse primer 5′-CAGCTCGTTCTCAAGCAGCAT-3′. For GRP (NM_002091), GRP-R (NM_005314), and control glyceraldehyde 3-phosphate dehydrogenase (GAPDH)-specific oligonucleotide primers were used as previously [13].

### Immunohistochemistry

For AKT2 staining, xenografts were fixed in formalin overnight and embedded in paraffin wax. Tumor sections (5 µm) were mounted on glass slides and staining was performed as previously described [5].

### Liver metastasis model

Male athymic nude mice (4–6 weeks old) were maintained as described [4]. All studies were approved by the Institutional Animal Care and Use Committee at the Vanderbilt University Medical Center (Protocol is M/09/304) and were conducted in accordance with guidelines issued by the National Institutes of Health. BE(2)-C cells were stably transfected with shCON or shAKT2 plasmid DNA. Mice were anesthetized with isofluorane, and a small left flank incision was made to exteriorize the spleen. Viable shCON or shAKT2 cells (2×10^6^ cells/100 µl HBSS) were injected into the splenic capsule using a 27-gauge needle. The spleen was returned to the abdominal cavity, and the surgical wound was closed with metal clips. Tumor growth was observed bi-weekly with Illumatool TLS (Lightools Research, Encinitas, CA). At the time of sacrifice, livers were excised, weighed, and fixed in formalin for further assessment.

### Statistical analysis

For *in vitro* experiments, conditions were compared using the student's paired t test. One-way analysis of variance (ANOVA) was performed for multiple comparisons. *In vivo* experiments were analyzed as described previously [4]. Body weight was analyzed using ANOVA for a two-factor experiment with repeated measures on time. For all experiments, *p*<0.05 was considered significant.

## Results

### Downregulation of GRP/GRP-R reduced expressions of N-myc

**Figure 1:**
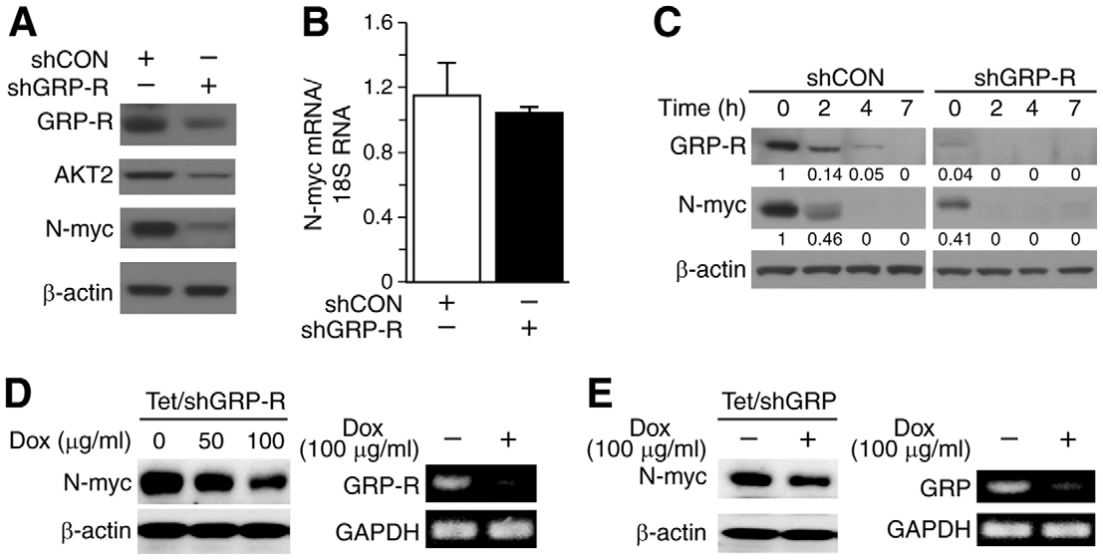
GRP/GRP-R regulated N-myc expression. (**A**) N-myc and AKT2 expression in BE(2)-C/shCON and BE(2)-C/shGRP-R cells by Western blotting. (**B**) *MYCN* mRNA levels, measured by real-time QRT-PCR, remained relatively unchanged. (**C**) Cells were serum-starved for 24 h and then re-plated in fresh RPMI media with 10% FBS. Decreased GRP-R expression in shGRP-R cells when compared to shCON cells was confirmed. N-myc expression was also decreased in shGRP-R cells at 0 and 2 h. Protein levels were quantified by densitometric analysis values indicated each band. (**D**) Inducible GRP-R silencing BE(2)-C/Tet/shGRP-R cells were treated with doxycyclin for 48 h, and then N-myc expression was analyzed by Western blotting. N-myc protein level was correspondingly decreased with GRP-R silencing (*left*); Decreased GRP-R mRNA was confirmed with RT-PCR (*right*). (**E**) Similar to GRP-R silencing, inducible GRP silencing BE(2)-C/Tet/shGRP cells were treated with doxycyclin for 48 h, and then N-myc expression was assessed with Western blotting (*left*). Inducible knockdown of GRP mRNA was confirmed with RT-PCR (*right*).

We have previously reported that silencing GRP-R, a G-protein coupled receptor (GPCR), attenuated AKT signaling in neuroblastoma cells [13]. Moreover, targeting GRP-R specifically suppresses the expression of the AKT2 isoform [13]. The role of GPCRs in the regulation of N-myc via PI3K/AKT pathway modulation has not been studied yet. So we wanted to examine whether GRP/GRP-R signaling regulates N-myc expression in *MYCN* amplified BE(2)-C cells. We examined N-myc expression in GRP-R silencing cells, and found that N-myc expression was significantly reduced in comparison to control cells (Fig. 1A). However, the mRNA levels of *MYCN*, as measured by real-time PCR, were not appreciably affected by GRP-R silencing (Fig. 1B). In order to exclude the effects of regulation of N-myc expression by cell cycle, the cells were synchronized by serum-starvation for 24 h, then re-fed in RPMI media with 10% FBS. The expression of N-myc protein in BE(2)-C/shGRP-R cells was significantly decreased when compared to that in shCON cells at 0 h, and completely degraded after 2 h (Fig. 1C). Our results suggest that GRP-R activation of cell signaling regulates endogenous N-myc expression at post-translational level. In order to exclude the non-specific effects of stable GRP-R silencing, we established a doxycycline-inducible silencing system in BE(2)-C cells [BE(2)-C/Tet/shGRP-R], in which GRP-R can be conditionally knocked down by doxycycline treatment. N-myc expression was significantly decreased in a dose-dependent manner after doxycycline-induced GRP-R silencing (Fig. 1D). Furthermore, using another doxycycline-inducible system for silencing GRP, a specific ligand of GRP-R, we assessed the effects of GRP silencing on N-myc expression in BE(2)-C cells. Our results were similar to those from shGRP-R inducible system, demonstrating the downregulation of N-myc expressions by GRP/GRP-R silencing (Fig. 1E). Hence, our data indicate that GRP/GRP-R signaling regulates N-myc at a post-translational level in neuroblastoma cells.

### AKT2 mediated N-myc expression in neuroblastoma cells

**Figure 2:**
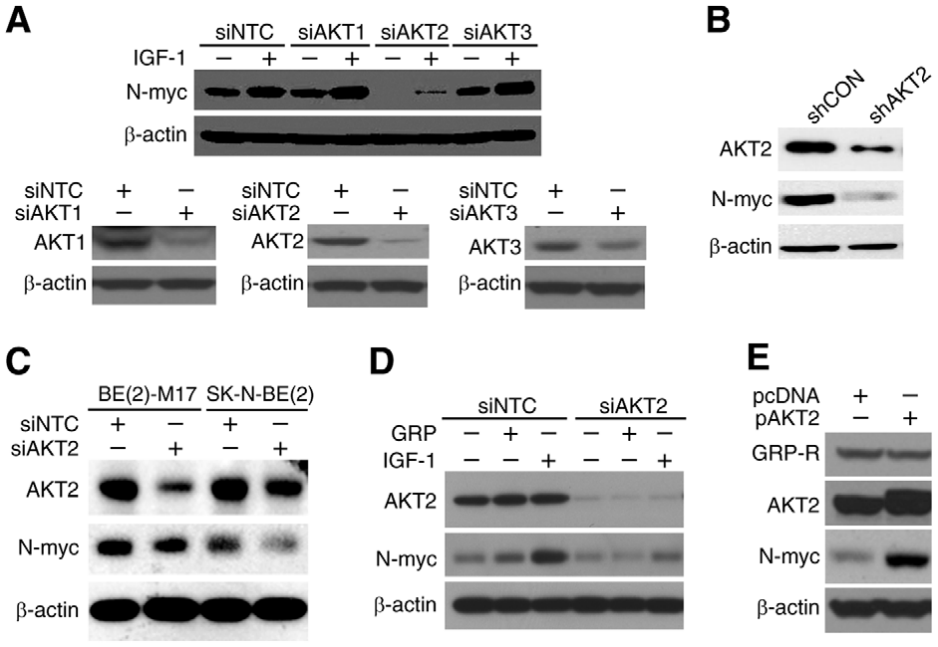
AKT2 regulated N-myc expression. (**A**) BE(2)-C cells were transfected with siRNA pools specifically targeting siAKT1, siAKT2, and siAKT3. Cells were serum-starved for overnight 48 h after transfections, and then stimulated with IGF-1 (100 nM) for 2 h. N-myc expression was examined by Western blotting. Notably, only siAKT2 decreased N-myc expression. Knockdown efficiency of each isoform was confirmed at 48 h post-transfection. (**B**) N-myc expression was examined in stably-transfected BE(2)-C/shCON and BE(2)-C/shAKT2 cells using Western blotting. N-myc expression was reduced in AKT2 stably knockdown cells. (**C**) BE(2)-M17 and SK-N-BE(2) cells were transiently transfected with AKT2 siRNA (siAKT2) pool. Proteins were extracted from cells 72 h after transfected with siAKT2. N-myc expression was downregulated in siAKT2 cells as examined by Western blot analysis. (**D**) BE(2)-C cells were transiently transfected with siAKT2 for 24 h, then serum-starved for another 24 h. They were then treated with either GRP (100 nM) or IGF-1 (100 nM) for 2 h. Western blotting showed suppression of N-myc by siAKT2 despite stimulation with GRP or IGF-1. (**E**) BE(2)-C cells were transiently transfected with the AKT2 overexpression plasmid pcDNA-Myr-AKT2 (pAKT2) or a control plasmid pcDNA for 48 h. AKT2 overexpression was confirmed by Western blot analysis. N-myc expression increased, but GRP-R was not changed in AKT2 overexpressed cells.

N-myc, a strong predictor of poor outcomes in patients with neuroblastoma, acts as a downstream effector in PI3K/AKT mediated oncogenic transformations in neuroblastoma cells *in vitro* and *in vivo* [17]. A recent study further demonstrated that anti-angiogenic efficacy of NVP-BEZ235, which is a dual inhibitor of PI3K and mTOR, depended critically on *MYCN in vitro* and *in vivo* [18]. Our results showed that GRP-R silencing resulted parallel decreased expression of AKT2 and N-myc (Fig. 1A), however, whether AKT directly effects N-myc expression in neuroblastoma cells has not been determined yet. In order to test this, we examined the expression of N-myc in cells transiently transfected with siRNA pools against AKT1, AKT2 or AKT3, respectively, with insulin-like growth factor (IGF-1) stimulation, as it has been reported that IGF-1 induces *MYCN* transcription [17], [19]. Our result displayed that silencing of AKT2 caused the most significant downregulation of N-myc expression in comparison to AKT1 or AKT3 silencing (Fig. 2A, *top*). Silencing of each isoform with siRNA pool was confirmed before IGF-1 treatment using Western blot analysis (Fig. 2A, *bottom*). To confirm whether AKT2 regulates N-myc expression, we used shRNA-mediated stably transfected AKT2 silenced cells [BE(2)-C/shAKT2] and observed a similar effect on N-myc protein levels when compared to control cells [BE(2)-C/shCON] (Fig. 2B). Similar results in two additional *MYCN* amplified neuroblastoma cell lines, BE(2)-M17 and SK-N-BE(2), confirmed that AKT2 regulation of N-myc is not a cell-line specific effect, and universally observed in different neuroblastoma cells lines (Fig. 2C).

We previously reported that GRP stimulates PI3K/AKT signaling pathway [3]. Here, we speculated that GRP could induce N-myc expression via AKT2. We treated AKT2 silenced neuroblastoma cells with or without GRP (100 nM) for 2 h after serum-starvation overnight, and IGF-1 (100 nM) was used as positive control. Our results showed that N-myc expression by exogenous GRP treatment was completely attenuated in BE(2)-C/siAKT2 cells as demonstrated by Western blotting (Fig. 2D). Meanwhile, AKT2 overexpression upregulated N-myc protein levels without affecting GRP-R expression (Fig. 2E), indicating that AKT2 is upstream of N-myc, but a downstream target of GRP-R. Taken together, these observations confirm that AKT2 is a critical regulator of N-myc expression in neuroblastoma cells.

### Silencing AKT2 decreased the tumorigenic potential of neuroblastoma cells *in vitro*

**Figure 3:**
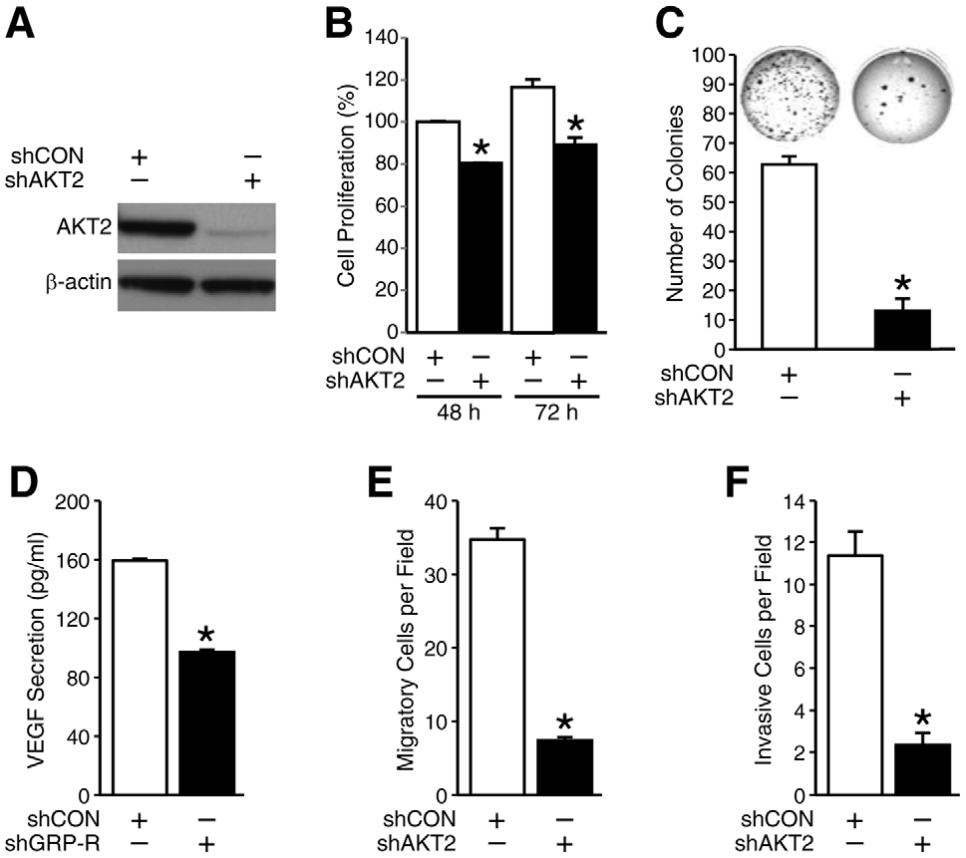
AKT2 regulated the tumorigenic potential of neuroblastoma cells *in vitro*. (**A**) AKT2 expression was measured in BE(2)-C/shCON and BE(2)-C/shAKT2 cells by Western blotting and consistent knockdown of AKT2 was confirmed. (**B**) BE(2)-C/shCON and shAKT2 cells were replated at 1×10^4^ cells per well in 96-well plates and cell proliferation was measured after culturing for 48 and 72 h. (**C**) Anchorage-independent growth was assessed by soft agar colony assay in BE(2)-C/shCON and shAKT2 cells. Cells were plated in soft agar (2×10^3^ cells/well) for 3 weeks and then photographed after staining with 0.005% of crystal violet. Colony growth was significantly inhibited in the cells treated with shAKT2. Bar graph represents the quantitative assessment of colony growth. (**D**) VEGF secretion was measured in BE(2)-C/shCON and shAKT2 cells. Cells (3×10^5^ cells/well) were plated in 6-well plate and cultured for 24 h, then the supernatants were collected for VEGF secretion by ELISA. (**E**) The number of migratory cell decreased in AKT2 silenced cells. BE(2)-C/shCON and shAKT2 cells plated in collagen type I-coated transwell plates and incubated for 6 h, the migrated cells were fixed and stained with DAPI and counted. (**F**) The number of invasive cell decreased in AKT2 silenced cells. BE(2)-C/shCON and shAKT2 cells plated in Matrigel coated transwells and incubated for 48 h. The invasive cells were fixed and stained with DAPI and counted. Data represent mean ± SEM.; * = *p*<0.05 vs. shCON.

AKT isoforms are known to mediate the acquisition of multiple hallmarks of cancer by tumor cells [20]. AKT2 mediates tumor cell migration and invasion of breast cancer cells [11]. However, much is unknown about its role in neuroblastoma tumorigenesis. To clarify the roles of AKT2 on cell proliferation, anchorage-independent growth, motility and angiogenesis in neuroblastoma, we used shRNA-mediated stably AKT2 silenced BE(2)-C/shAKT2 and control shCON cells (Fig. 3A) and performed functional assays *in vitro*. Our results demonstrated that AKT2 silencing decreased cell proliferation by 20% and 30% at 48 h and 72 h, respectively (Fig. 3B). The soft agar colony number was inhibited by 84% in comparison to control cells (Fig. 3C). Our results indicated that AKT2 silencing inhibited the cell anchorage-independent growth *in vitro* and decreased the potential to metastasize to secondary sites *in vivo*. Interestingly, VEGF secretion in the cell culture supernatant of BE(2)-C cells with AKT2 silencing was decreased by 50% when compared to that in cell culture supernatant from control cells (Fig. 3D), implicating a role for AKT2 isoform in tumor-mediated angiogenesis. Moreover, both migration and invasion of AKT2 stably silenced neuroblastoma cells were decreased by approximately 80% when compared to controls (Figs. 3E and F). Therefore, we conclude that AKT2 has critical oncogenic roles in neuroblastoma cell growth and motility, as well as VEGF secretion *in vitro*.

### Silencing AKT2 decreased neuroblastoma liver metastasis *in vivo*

**Figure 4:**
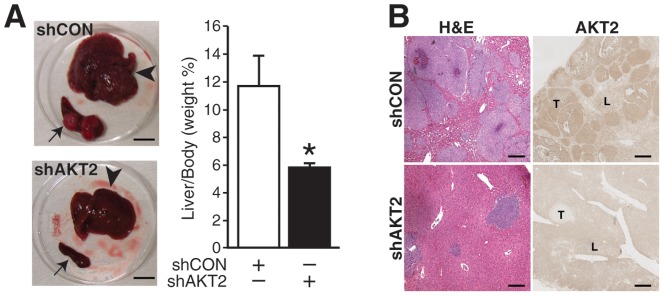
AKT2 modulated neuroblastoma liver metastasis. (**A**) Representative photographs of splenic primary tumors (*arrow*s) and liver metastases (*arrowheads*) from mice injected with BE(2)-C/shCON and BE(2)-C/shAKT2 cells. Liver metastases were fewer in mice injected with BE(2)-C/shAKT2 cells (*bottom*). Bar scale is 10 mm. Bar graph represents the quantitative analysis of liver weight relative to mice body weight (* = *p*<0.05 vs. shCON). (**B**) Representative H&E-stained liver sections from mice injected with BE(2)-C/shCON and BE(2)-C/shAKT2 (*left panels*). Immunohistochemical staining of AKT2 in liver sections from mice injected with BE(2)-C/shCON and BE(2)-C/shAKT2 cells (*right panels*). (“L” = liver, “T” = tumor; bar scale is 50 µm).

Since AKT2 played an important role in the anchorage-independent growth and cell motility of neuroblastoma cells (Fig. 3), we speculated that AKT2 might regulate tumor metastasis *in vivo*. In order to confirm this hypothesis, we performed animal studies using an intrasplenic injection liver metastasis murine model established in our laboratory [13]. We injected shRNA mediated stably transfected BE(2)-C/shCON or BE(2)-C/shAKT2 cells into the spleen of mice. In the control group, BE(2)-C/shCON induced large primary tumor mass in spleen, and secondary metastatic lesions in liver within three weeks post tumor cell inoculation. In contrast, BE(2)-C/shAKT2 injections resulted in smaller splenic primary tumors. Interestingly, BE(2)-C/shAKT2 produced significantly fewer liver metastases (Fig. 4A, *left top*), and the liver appeared grossly normal and healthy. Histological assessment showed increased liver tumor infiltrations in the BE(2)-C/shCON group when compared to those in the BE(2)-C/shAKT2 group, which demonstrated a more normal architectural appearance with few tumor foci (Fig. 4A, *left bottom*). The liver weights of mice injected with BE(2)-C/shAKT2 cells resembled that of normal mice. In contrast, livers of mice injected with BE(2)-C/shCON cells, endogenously expressing abundant AKT2, were significantly larger and nearly twice as heavy than those of BE(2)-C/shAKT2 group (Fig. 4A, *right*). Hematoxylin and Eosin staining of the livers showed numerous, densely packed neuroblastoma metastases in the BE(2)-C/shCON group, whereas rare micrometastases were observed in the livers from the BE(2)-C/shAKT2 group (Fig. 4B). Immunohistochemical staining confirmed that AKT2 was intensively expressed in BE(2)-C/shCON tumors, while it was significantly attenuated in BE(2)-C/shAKT2 tumors. Therefore, our findings indicate that AKT2 is critically involved in neuroblastoma liver metastasis *in vivo*.

## Discussion

In neuroblastoma, >50% of patients have metastatic disease at diagnosis, and thus creating major challenges for treatment and cure. Moreover, ‘high-risk’ group of neuroblastomas often relapse despite initial response to therapies. Frequently, tumors acquire drug resistance or aggressive phenotypes through the selection of rare resistant clones from heterogeneous tumor environment, which can result in major clinical obstacles in the treatment of neuroblastoma [15]. Thus, a better understanding of the mechanisms of signaling pathways that contribute to metastasis may be valuable in the development of novel therapies.

Oncogene *MYCN* is amplified and overexpressed in 25% of neuroblastoma patients [14], [21], and correlates to poor outcomes in older children [16]. PI3K/AKT pathway utilizes N-myc as a critical downstream effector to enhance tumorigenicity of neuroblastoma cells *in vitro* and *in vivo* [17], [18]. In this study, we found that silencing AKT2, but not AKT1 or AKT3 suppresses N-myc expression in neuroblastoma cells. This is a novel observation, implicating a specific AKT2 isoform as a critical regulator of N-myc in neuroblastoma cells. Interestingly, a recent study has shown that *MYCN* contributes to tumorigenesis, in part, by repressing miR-184, and increasing AKT2 expression, a direct target of miR-184 [22], and thereby indicating that AKT2 is a downstream target of N-myc. Overall, a positive regulatory loop might exist between the two oncogenic proteins, AKT2 and N-myc in human neuroblastoma cells, which contributes crucially to tumorigenicity. Moreover, we also report, for the first time, that N-myc expression can be regulated at the post-translational level by GRP-R, a GPCR involved in neuroblastoma tumorigenesis. Since, GRP-R silencing specifically inhibited the expression of AKT2 isoform, but not AKT1 or AKT3, we can further conclude that GRP-R-mediated regulation of N-myc expression in neuroblastoma cells is AKT2-dependent.

We previously showed that a ratio of phosphorylated AKT to PTEN levels correlates with degree of differentiation in neuroblastomas; an increased ratio of AKT to PTEN expression was found in more undifferentiated tumors [5]. Of the three AKT isoforms, AKT2 has been implicated more frequently in cancers [9], [11], [12], [20]. Consistent with other cancer cell types, we report, for the first time, that AKT2 is critical for neuroblastoma progression. AKT2 plays an important role in human neuroblastoma cells as a downstream target of GRP/GRP-R and regulates neuroblastoma cell proliferation, anchorage-independent growth, migration and invasion *in vitro*, implicating AKT2 in multiple aspects of neuroblastoma initiation and progression. Furthermore, targeting AKT2 decreased VEGF secretion by neuroblastoma cells demonstrating a crucial role for this isoform in tumor cell-mediated angiogenesis. Correspondingly, our murine model demonstrated that silencing AKT2 decreased metastasis to the liver and formation of secondary lesions in comparison to mice injected with control neuroblastoma cells with high endogenous expression of AKT2. The oncogenic role of AKT2 demonstrated in this study may provide a possible explanation as to why AKT activation has been shown to be a predictor of poor outcome in patients with neuroblastoma.

In summary, our findings further support the notion that GRP/GRP-R is a promising therapeutic target in the treatment of clinically aggressive neuroblastomas. Moreover, GRP-R modulates N-myc expression in neuroblastoma cells by AKT2 isoform, but not the AKT1 or AKT3. Targeting GRP/GRP-R/AKT2 would be advantageous in developing a novel therapeutic option for aggressive and undifferentiated neuroblastomas with a high propensity for metastasis.
